# Predicting Sodium-Ion Battery Performance through Surface Chemistry Analysis and Textural Properties of Functionalized Hard Carbons Using AI

**DOI:** 10.3390/ma17174193

**Published:** 2024-08-24

**Authors:** Walter M. Warren-Vega, Ana I. Zárate-Guzmán, Francisco Carrasco-Marín, Guadalupe Ramos-Sánchez, Luis A. Romero-Cano

**Affiliations:** 1 Grupo de Investigación en Materiales y Fenómenos de Superficie, Departamento de Biotecnológicas y Ambientales, Universidad Autónoma de Guadalajara, Av. Patria 1201, C.P., Zapopan 45129, Mexico; 2 Unidad de Excelencia Química Aplicada a Biomedicina y Medioambiente, Materiales Polifuncionales Basados en Carbono (UGR-Carbon), Departamento de Química Inorgánica, Facultad de Ciencias, Universidad de Granada (UEQ-UGR), 18071 Granada, Spain; 3 Departamento de Ingeniería de Procesos e Hidráulica, Universidad Autónoma Metropolitana, Unidad Iztapalapa, Av. San Rafael Atlixco 186, Mexico City 09340, Mexico

**Keywords:** machine learning, artificial neural network, MATLAB-Simulink, artificial intelligence, energy storage mechanism

## Abstract

Traditionally, the performance of sodium-ion batteries has been predicted based on a single characteristic of the electrodes and its relationship to specific capacity increase. However, recent studies have shown that this hypothesis is incorrect because their performance depends on multiple physical and chemical variables. Due to the above, the present communication shows machine learning as an innovative strategy to predict the performance of functionalized hard carbon anodes prepared from grapefruit peels. In this sense, a three-layer feed-forward Artificial Neural Network (ANN) was designed. The inputs used to feed the ANN were the physicochemical characteristics of the materials, which consisted of mercury intrusion porosimetry data (S_Hg_ and average pore), elemental analysis (C, H, N, S), I_D_/I_G_ ratio obtained from RAMAN studies, and X-ray photoemission spectroscopy data of the C_1s_, N_1s,_ and O_1s_ regions. In addition, two more inputs were added: the cycle number and the applied C-rate. The ANN architecture consisted of a first hidden layer with a sigmoid transfer function and a second layer with a log-sigmoid transfer function. Finally, a sigmoid transfer function was used in the output layer. Each layer had 10 neurons. The training algorithm used was Bayesian regularization. The results show that the proposed ANN correctly predicts (R^2^ > 0.99) the performance of all materials. The proposed strategy provides critical insights into the variables that must be controlled during material synthesis to optimize the process and accelerate progress in developing tailored materials.

## 1. Introduction

The search for sustainable and efficient energy storage devices has become increasingly important, especially as the demand for mobile electronic devices continues growing. Currently, lithium-ion batteries dominate the market; however, there is a pressing need to find viable alternatives that can provide similar or improved performance. Sodium-ion batteries have emerged as a promising alternative, demonstrating similar mechanisms and behaviors in energy storage and generation compared to lithium-ion batteries [1,2]. Sodium-ion batteries have recently received extensive research and attention recently due to their similarity to lithium-ion batteries and the abundance of sodium resources. Apart from the large-scale energy storage, they also show potential in the field of medium-range (160–240 miles) electric vehicles (EVs) [3].

One of the significant challenges in the development of sodium-ion batteries is the implementation of effective anode materials. Carbon materials, particularly those that are biomass-derived, offer a low-cost and sustainable option. The properties of these carbon materials—such as their textural and chemical characteristics—have a direct impact on the electrochemical reactions involved in energy storage and generation [4,5]. Various studies have proposed different carbon materials for this purpose, highlighting the need for extensive research to understand the relationship between material properties and battery performance [6,7,8]. In this sense, we highlight the work presented by Romero-Cano et al. (2019) [4], in which a surface functionalization strategy is applied to increase the reversible capacity of anode materials for sodium-ion batteries based on grapefruit peels. It was demonstrated that the functionalization process has a deep effect on the accessibility of the electrolyte to the porosity of the materials and the sodium diffusion coefficient; also, it affects the reactivity of the surface, allowing stable and more reversible sodium intercalation. Considering that the improved reversible capacity is mainly related to the structural surface disorder caused by the introduction of nitrogen functional groups, it is proposed that the characterization of the materials is closely related to the performance of the batteries, so it would be possible to use this information in combination with advanced chemometric techniques to predict the electrochemical behavior of hard carbon materials.

To understand the efficiency of these processes, artificial intelligence (AI), particularly Artificial Neural Networks (ANNs), can be employed. ANNs are capable of estimating, predicting, and classifying based on inputs, which makes them suitable for modeling complex phenomena such as energy storage and generation in batteries [9,10,11,12]. By using AI, is possible to establish a system that not only understands the underlying mechanisms but also proposes tailor-made materials for optimized performance.

In the present research, we aim to develop an AI algorithm that can establish a correlation between the textural and chemical properties of carbon materials and their impact on the performance of sodium-ion batteries. Specifically, the energy storage capacity will be evaluated based on the textural properties and surface chemistry of the materials in repeated cycles at different rates. Neural networks have been previously reported to predict various material properties, such as porosity [13], surface topography [14], viscosity [15], and mechanical properties [16]. This method is effective because it allows for the description of a phenomenon under defined conditions. Once trained, a neural network model can be used to predict the performance of sodium-ion batteries, providing valuable insights into the energy storage mechanisms. By predicting the performance of these batteries based on the properties of carbon materials, we can better understand and optimize the storage mechanisms. Moreover, the trained neural network model can provide insights that lead to the design of new materials with improved energy storage capabilities, paving the way for more efficient and sustainable battery technologies.

## 2. Materials and Methods

### 2.1. Preparation and Characterization of Functionalized Hard Carbons

The synthesis involves washing and drying biomass at 110 °C for 12 h, followed by pyrolysis in a tubular stainless-steel oven up to 600 °C for 1 h under a nitrogen atmosphere. The material obtained was labeled as GPC. Carbon material functionalization was then carried out using NaOH and citric acid treatments, followed by further modification with urea or melamine. Initially, carbon materials are washed with NaOH solution (0.1 M), followed by citric acid (0.6 M) treatment to introduce carboxyl groups onto the surface, GPC-AC material. Subsequently, urea or melamine modification is performed using the incipient wetness impregnation method at specific weight ratios: 0.5 for urea and 14.3 for melamine (GPC-AC/Urea or Melamine). The resulting samples are labeled as GPC-AC-U for urea-modified samples and GPC-AC-M for melamine-modified samples. Finally, the materials are dried and thermally treated under the same conditions as the initial pyrolysis process [4].

Elemental analysis for all samples was obtained in a Thermo Finningan Flas EA1112 CHNS-O elemental analyzer (Thermo Fisher Scientific, Waltham, MA, USA). A Thermo Scientific DRX RAMAN micro spectrophotometer was used at a laser wavelength of 780 nm and a power of 4 mW in a range of 200–2400 cm^−1^ to evaluate the degree of graphitization. X-ray photoemission spectroscopy (XPS) was also used to characterize the surface chemistry using a K-Alpha ± Thermo Scientific spectrometer (Thermo Fisher Scientific, Waltham, MA, USA). Spectra were acquired with an Al Kα X-ray source operating at 150 W, 15 kV, and 10 mA under a base pressure of 3 × 10^−8^ Torr. Wide-scan spectra covered an energy range of 0–1350 eV with an energy step of 80 eV and a step size of 1 eV. High-resolution spectra were obtained with an energy step of 40 eV and a step size of 0.1 eV. Each spectral region of interest (C_1s_, O_1s_, and N_1s_) was scanned multiple times to ensure adequate signal-to-noise ratios. Peak positions and areas were determined after background signal correction, and resulting spectra were fitted using Lorentz and Gauss plots (Voigt function). The peak assignment was based on recent literature. Finally, mercury intrusion porosimetry studies were carried out in a Quantachrome Poremaster to obtain pore volume, total pore area accessible to mercury, and pore size distribution curves for the larger macropores and mesopores.

### 2.2. Electrochemical Experiments

The anodes were prepared using the functionalized hard carbons following standard procedures [17,18,19]. Electrochemical experiments were conducted in an ECC Combi cell (EL-CELL) using cleansed sodium foil as a counter and reference electrode. The electrolyte was prepared with equivalent volumetric amounts of Ethylene Carbonate (EC), Propylene Carbonate (PC), and Dimethyl Carbonate (DMC) containing 1 M NaPF_6_. The electrolyte was impregnated in a glass fiber separator (Whatman, Maidstone, UK) and sandwiched between the carbon-containing electrode and sodium foil. All measurements were performed in a multipotentiostat/galvanostat VMP3 (Biologic Science Instruments (Biologic Science Instruments, Claix, France)). For charge/discharge experiments, the upper voltage cutoff was set at 3 V and the discharge cutoff at 0.003 V. Rate performance testing involved full discharges at various rates (C/10, 1C, 5C, 10C, and C/10) based on graphite’s theoretical capacity. Electrochemical characterization was conducted on newly assembled cells after a 2 h stabilization period.

### 2.3. Artificial Neural Network (ANN) to Study the Performance in Sodium-Ion Batteries

**Figure 1:**
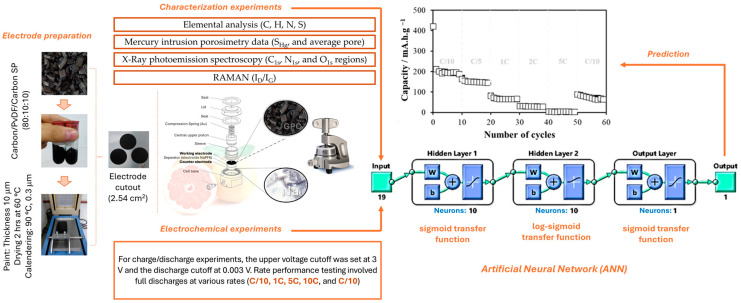
Graphic description of the proposed ANN’s experimental methodology and architecture to predict sodium-ion batteries’ performance.

MATLAB software (Matlab R2024a) was used to obtain a three-layer feed-forward artificial neural network. The inputs used to feed the ANN were the characterization of the materials that consisted of mercury intrusion porosimetry data (S_Hg_, and average pore), elemental analysis (C, H, N, S), I_D_/I_G_ ratio obtained from RAMAN studies, and X-ray photoemission spectroscopy data of C_1s_, N_1s_, and O_1s_ regions. In addition to this information, two more inputs were added: the cycle number and the C-rate applied, to have a total of 19 inputs. The architecture consists of the first hidden layer with a sigmoid transfer function, in the second layer a log-sigmoid transfer function, and finally, in the output layer, a sigmoid transfer function. The neurons used in each layer were 10 neurons. The dataset used to implement the algorithm of the ANN consisted of 4720 data points, which were randomly divided into three sets: training (70%), validation (15%), and test (15%). The scheme of the ANN chosen was based on its performance results considering the Mean Square Error (MSE) and the fit of the data to the regression by its correlation coefficient (R) [20]. The training algorithm used was the Bayesian regularization, which permits evaluating a non-linear regression and estimating a model that is more robust than standard networks [21]. The architecture of the ANN is presented in Figure 1.

## 3. Results and Discussion

### 3.1. Physical-Chemical Characterization of Hard Carbons and Development of ANN Algorithm

The discussion of the physicochemical characterization of the materials in depth has been previously reported [4]. For the purposes of the development of the ANN and the study of the energy storage mechanism using artificial intelligence, Table 1 compiles the characterization of the surface chemistry and textural properties of the synthesized carbon materials. It is observed that depending on the type of preparation, different amounts of nitrogen bound to the structure are obtained in a range of 1.3 %wt to 4.7 %wt. Consequently, the materials present many topological defects in the graphene layers, which have been evaluated from the relationship between the intensities of the D and G bands (I_D_/I_G_) of the RAMAN spectra [22]. The results show a linear relationship with the amount of nitrogen in the material. It is concluded that the defects in the structure are induced by the nitrogen functionalities added to the carbon materials. These have been characterized by XP spectroscopy, highlighting that the unfunctionalized material (GPC) mainly contains -C=O, -COO^–^, and NH_3_^+^ groups, characteristic of materials of cellulosic origin. After the functionalization of the carbon with the acidic agent (GPC-AC), -COOH groups are observed attributable to the functionalization with citric acid. These groups serve as anchoring sites for the samples functionalized with urea (GPC-AC-U) and melamine (GPC-AC-M), mainly presenting -NH, -CN, and -NH_2_ groups [23].

Finally, mercury intrusion porosimetry studied the hard carbons’ textural properties to complete the characterization. In all cases, low surface areas are observed (3–11 m^2^ g^−1^) attributable to cellulosic materials synthesized at low temperatures [24]. The materials can be considered to be essentially meso- and macroporous with a mean pore diameter with respect to an area of 0.03 mm. Due to functionalization, pore volumes are modified. The main characteristic corresponds to a decrease in volume, indicating constrictions at the entrance of the mesopores making them less accessible.

This information along with the energy storage capacity at different charging cycles and rates were used as inputs and outputs to develop the ANN.

**Figure 2:**
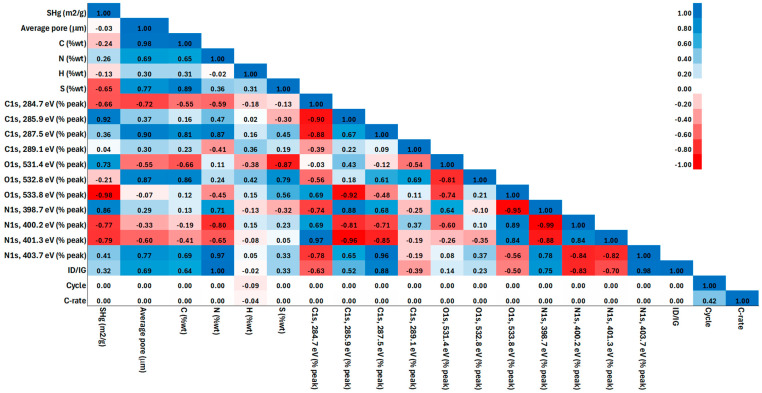
Pearson’s correlation matrix of the select variables.

Figure 2 shows the Pearson correlation matrix of the variables fed as an input to the ANN. It is observed that variables such as S_Hg_ and O_1s, 533.8 eV_ (% peak) exhibit near-uniform distributions, whereas variables like N_1s, 398.7 eV_ (% peak), and N_1s, 403.7 eV_ (% peak) show a more skewed distribution. The preprocessing phase has considered these distributions to ensure the model can effectively learn from the data. The correlation matrix reveals several key insights: For example, S_Hg_ and C_1s, 285.9 eV_ (% peak) have a high positive correlation (0.92), indicating that these variables likely contribute similarly to the model’s predictions. Similarly, I_D_/I_G_ and N_1s, 403.7 eV_ (% peak) also exhibit a strong positive correlation (0.98), suggesting that these features are crucial for capturing the underlying patterns in the data. On the other hand, there are also significant negative correlations, such as between O_1s, 533.8 eV_ (% peak), and S_Hg_ (−0.98), which indicate that these variables have opposite effects on the output and are vital for the model’s accuracy.

The feature importance analysis based on the Pearson correlation coefficient has been instrumental in guiding the selection of input parameters. Based on the above, for the construction of the ANN, the variables with both strong correlations (to capture direct relationships) and those with low correlations (to ensure the model has access to diverse information sources) were used. This approach helps in reducing redundancy while maintaining the model’s ability to generalize, as evidenced by the robust performance metrics observed during cross-validation.

It is important to mention that, although there are variables with strong collinearity, their incorporation into the ANN model is essential to capture the complexity of the sodium storage mechanism in carbon anodes. It is observed that the carbon content (variable: C) and the average pore diameter (variable: average pore) present a strong correlation, which is an expected phenomenon because as the carbon content increases, the material tends to have a more porous structure. This behavior is crucial for the functionality of the anode since optimized porosity improves the efficiency of the material. However, sodium storage not only depends on the porous structure but also on the arrangement of the disordered graphitic layers (Variable: I_D_/I_G_) and the functionalization of the material (variables: C_1s_, O_1s,_ and N_1s_) since the incorporation of nitrogen introduces topological defects and modifies the carbon surface (variables: S_Hg_, N, H, and S), improving the intercalation of Na ions and contact with the electrolyte. Therefore, although some variables are collinearly related, their inclusion in the model is justified because they represent different aspects of the storage mechanism that cannot be adequately captured if eliminated. These elements provide a complete view of how carbon functionalization and structure affect anode performance.

**Figure 3:**
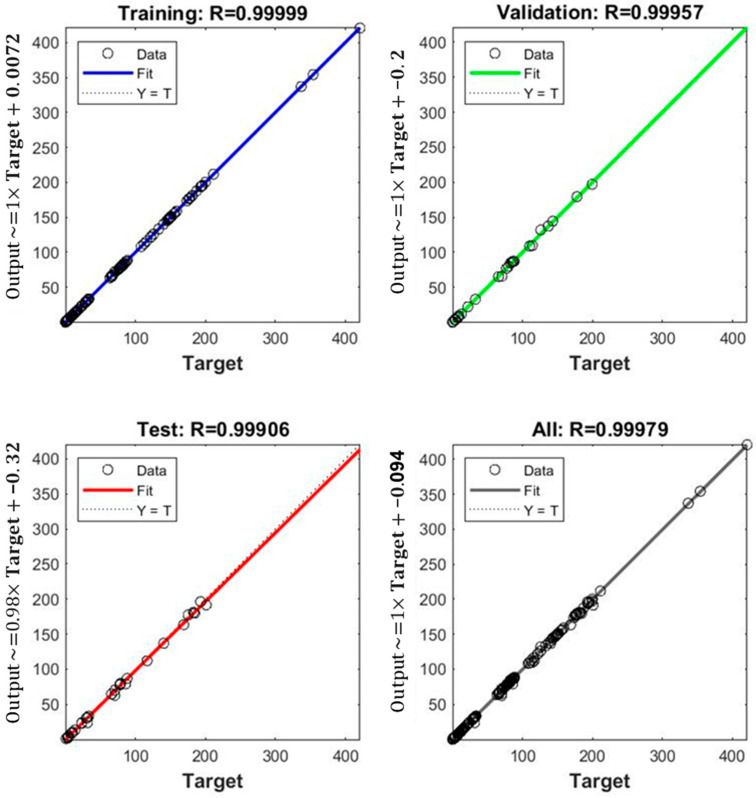
ANN training, validation, and test performance in its prediction of rate capability.

Figure 3 shows the adjustment of the electrochemical data to the algorithm in the three steps. The ANN model developed showed outstanding performance in the training, validation, and testing phases, reaching a coefficient of determination (R^2^) of 0.999 in each of these stages. These results indicate an excellent correlation between the network’s predictions and the values obtained experimentally, suggesting that the network was able to effectively capture the underlying relationships in the data. This high level of precision suggests that the selected architecture was highly effective in modeling the complex non-linear relationship between material characteristics and the target variable. The consistency in validation and testing results reflects good generalization ability, indicating that the model not only fits the training data well but is also effective in predicting new data.

### 3.2. Development of a Simulation Model in MATLAB-Simulink

Once the ANN was trained, a simulation model was created in MATLAB-Simulink, which is useful for predicting the performance of sodium-ion batteries based on the physicochemical characteristics described in Table 1. The model consisted of a “Custom Neural Network” block where the ANN code was loaded, to which a “Mux” block was connected as input that unites the inputs corresponding to the physicochemical characteristics of the sample to be analyzed (using a “Constant” block) and two “From Workspace” blocks, which were programmed with the variables “C-rate” and “Cycle” varying from C/10, C/5, 1C, 2C, and 5C every 10 cycles in order to predict the rate capability of hard carbons. Finally, the output of the “Custom Neural Network” block was connected to a “To Workspace” block.

**Figure 4:**
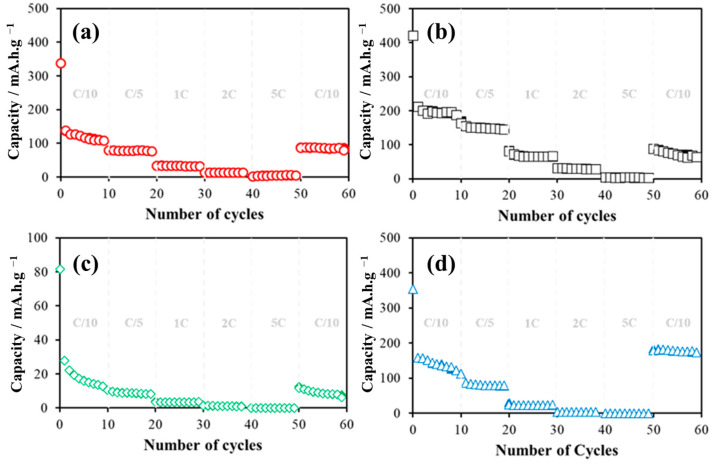
Rate capability performance of sodium-ion battery: (**a**) ○ GPC, (**b**) □ GPC-AC-U, (**c**) ◊ GPC-AC, and (**d**) ∆ GPC-AC-M. With solid fill for experimental data and without fill for ANN-predicted data.

The rate capability tests were conducted at various C-rates. The experimental and predicted data are presented in Figure 4. It is observed that the GPC samples initially show a high capacity, but they also experience rapid capacity decay during the initial cycles. This behavior suggests that functionalization helps stabilize the surface, leading to higher specific capacities. In contrast, samples stabilized with melamine, while showing greater stability compared to the GPC samples, still exhibit capacity loss across all C-rates. This indicates that melamine is less effective than urea. Moreover, the melamine-stabilized samples demonstrate a drastic reduction in area, resulting in a significant decrease in specific capacity from the very first cycle.

The simulation was carried out for all materials and the results obtained are presented in Figure 4. It is observed that the values predicted by the model do not present statistically significant differences (*p* < 0.05) with respect to the values obtained experimentally.

**Figure 5:**
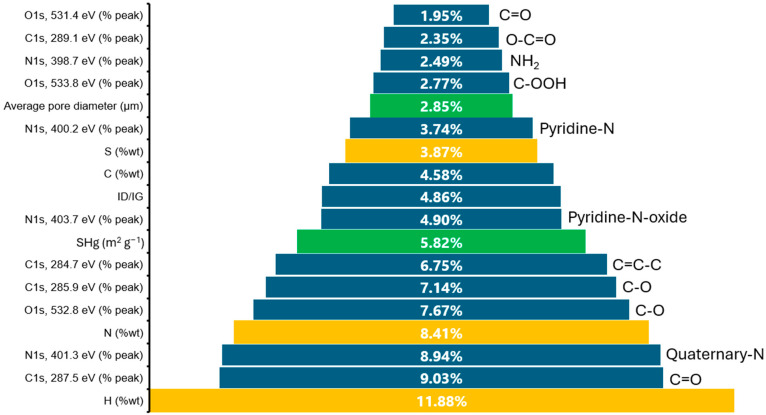
The relative contribution of each characterization parameter in the performance of anodes in sodium-ion batteries determined from the prediction using artificial neural networks: ■ surface chemistry, ■ porous texture, ■ elemental content.

To obtain information about the relative importance of each of the input variables with the prediction of the ANN, the magnitudes of the weights assigned to the connections between the inputs and the 10 interconnected neurons were studied. The relative contribution of each entry was obtained by dividing each of the assigned weights by the total sum of the weights for each neuron. The information in percentage is presented in Figure 5. When examining the normalized weights, it is observed that the factors that have the most significant influence on the response are the amount of nitrogen and hydrogen (%wt) present in the structure, representing 20.29% of the total participation, attributable to the fact that both elements act as an inhibitor of graphitization, since hydrogen and nitrogen occupy places in the carbon structure, making it difficult to form the ordered structure of graphite. Likewise, its presence induces defects in the carbon structure, generating disordered pseudo-graphitic plates.

It has been considered that the increase in the energy storage capacity of functionalized hard carbons is due to a linear relationship with the I_D_/I_G_ parameter, indicating that although other factors influence the mechanism, their participation can be considered despicable [4]. In this sense, the present work uses artificial intelligence to assign the corresponding weight to each of the stages and physicochemical characteristics, proposing that the sodium storage mechanism begins with a cation diffusion process within the porous network of the carbon; this step has a relative participation of 8.67%, corresponding to the physicochemical characterization of the material determined by mercury intrusion porosimetry. Once this process has happened, sodium ions are stored between the disordered graphitic layers of the material. This step has a relative participation of 29.02%, corresponding to the physicochemical characterization of the material determined by RAMAN spectroscopy and elemental analysis (H %wt, N %wt, S %wt). Finally, it is highlighted that the topological defects in the structure of the materials are induced by the functional groups introduced to the material, having a more significant contribution to the energy storage process in the following order: C=O (9.03%) > Quaternary-N (8.94%) > C-O (7.67%) > C=C-C (6.75%) > Pyridine-N-oxide (4.90%) > Pyridine-N (3.74%) > C-OOH (2.77%) > -NH_2_ (2.49%) > O-C=O (2.35%).

### 3.3. Comparison of Machine Learning Approaches for Performance Prediction in Energy Storage Systems

The estimation of performance in batteries is a time-series prediction problem affected by the complexity of battery reactions and environmental factors [25,26]. Data-driven methods, such as machine learning, have gained popularity in this field due to their ability to bypass the need for specific formulas or models and directly leverage input features. Notable examples include random forest regression [25], support vector machines (SVM) [27], Gaussian process regression (GPR) [28], ARIMA models [29], and recurrent neural networks (RNN) [30]. To overcome the limitations of RNNs in handling long time-series data, variants with gating mechanisms, such as LSTM [31] and GRU [32], have been developed, allowing the network to learn long-term dependencies and improve performance [33,34]. Bidirectional variants, such as BiGRU and BiLSTM, have also been introduced to account for past and future observations in non-linear systems [35].

A recent model, N-BEATS, which employs fully connected layers for time-series prediction, has shown potential as a regression model for SOC estimation [36,37]. Additionally, fusion models combining N-BEATS with deep neural networks (DNN) have demonstrated good robustness and generalization [38]. However, these methods often face challenges, such as long training times and issues with loss function convergence when increasing the number of layers.

In this context, the present research proposes a novel strategy using a three-layer Artificial Neural Network (ANN) to predict the performance of functionalized hard carbon anodes. The designed ANN architecture has proven highly accurate, with a coefficient of determination (R^2^) exceeding 0.99, indicating its reliability compared to the approaches mentioned. Furthermore, this strategy provides critical insights into the key variables that must be controlled during material synthesis to optimize anode performance.

## 4. Conclusions

It is possible to study the performance of sodium-ion batteries with the support of artificial intelligence. The development of a prediction model using a MATLAB-Simulink model based on an Artificial Neural Network (ANN) has managed to determine patterns between input variables associated with the characterization of the texture and surface chemistry of the materials used as anodes with the capacity charging of sodium-ion batteries. When examining the magnitude of the normalized weights returned by the ANN, it was observed that the factors that have the most significant influence on the response are the amount of nitrogen and hydrogen (%wt) present in the structure, representing 20.29% of the total participation, attributable because both elements act as an inhibitor of graphitization. From the ANN information, it has been possible to determine the relative contribution of each characterization parameter to the adsorption capacity of the material. The information presented contributes to a significant advance in the knowledge of using artificial intelligence in synthesizing custom materials.

## Figures and Tables

**Table 1:** Materials characterization of hard carbons based on textural, elemental, and surface chemistry analysis.

Input	GPC	GPC-AC	GPC-AC-U	GPC-AC-M
S_Hg_ (m^2^ g^−1^)	11.00	6.70	9.80	3.80
Average pore diameter (µm)	23.25	32.62	37.23	27.86
I_D_/I_G_	1.24	1.22	1.39	1.26
C (%wt)	76.26	80.20	81.75	79.17
N (%wt)	1.71	1.38	4.74	2.33
H (%wt)	1.74	1.99	1.82	1.81
S (%wt)	0.04	0.06	0.06	0.06
C_1s, 284.7 eV_ (% peak)	75	72	66	82
C_1s, 285.9 eV_ (% peak)	17	15	19	10
C_1s, 287.5 eV_ (% peak)	5	7	12	5
C_1s, 289.1 eV_ (% peak)	3	6	3	2
O_1s, 531.4 eV_ (% peak)	57	43	50	47
O_1s, 532.8 eV_ (% peak)	23	32	30	26
O_1s, 533.8 eV_ (% peak)	20	25	20	27
N_1s, 398.7 eV_ (% peak)	53	39	61	37
N_1s, 400.2 eV_ (% peak)	25	33	19	32
N_1s, 401.3 eV_ (% peak)	22	23	14	31
N_1s, 403.7 eV_ (% peak)	0	0	6	0

## Data Availability

The data that support the findings of this study are available from the corresponding author upon reasonable request.
